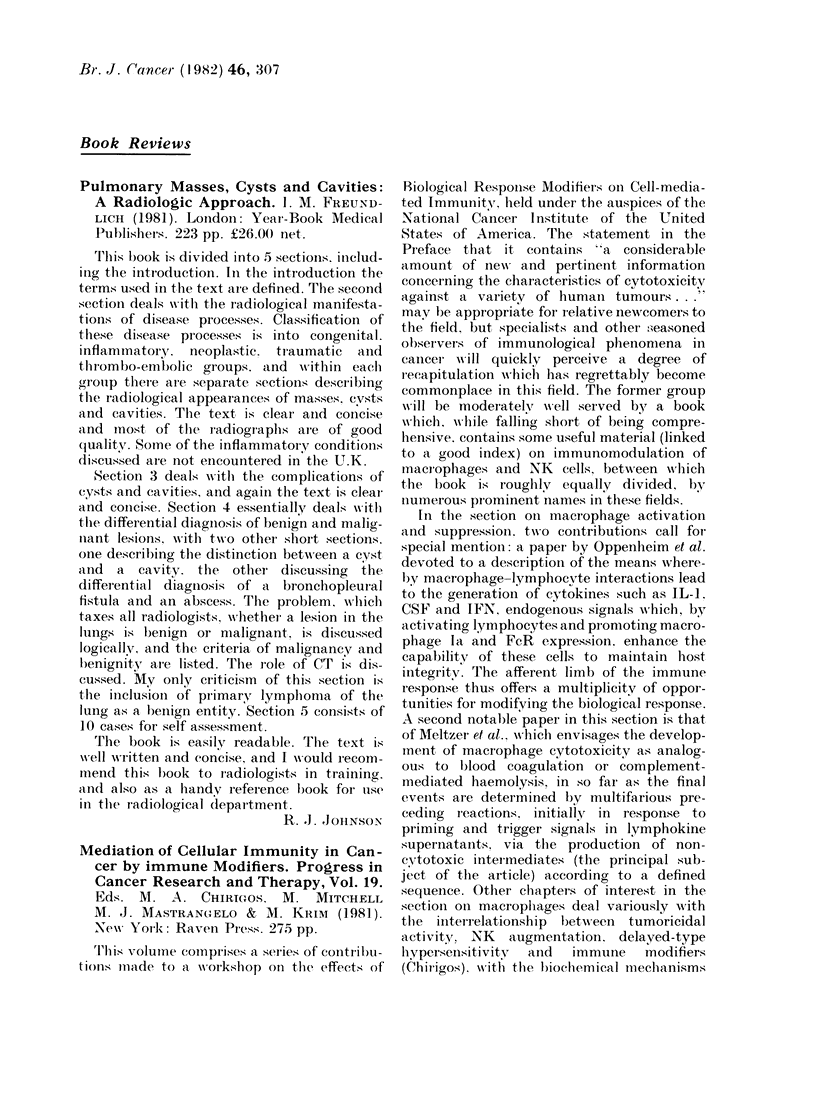# Pulmonary Masses, Cysts and Cavities: A Radiologic Approach

**Published:** 1982-08

**Authors:** R. J. Johnson


*Br. J. Cancer* (1982) **46**, 307

### *Book Reviews*

**Pulmonary Masses, Cysts and Cavities: A Radiologic Approach.** I. M. FREUNDLICH (1981). London: Year-Book Medical Publishers. 223 pp. £26.00 net.

This book is divided into 5 sections, including the introduction. In the introduction the terms used in the text are defined. The second section deals with the radiological manifestations of disease processes. Classification of these disease processes is into congenital, inflammatory, neoplastic, traumatic and thrombo-embolic groups, and within each group there are separate sections describing the radiological appearances of masses, cysts and cavities. The text is clear and concise and most of the radiographs are of good quality. Some of the inflammatory conditions discussed are not encountered in the U.K.

Section 3 deals with the complications of cysts and cavities, and again the text is clear and concise. Section 4 essentially deals with the differential diagnosis of benign and malignant lesions, with two other short sections, one describing the distinction between a cyst and a cavity, the other discussing the differential diagnosis of a bronchopleural fistula and an abscess. The problem, which taxes all radiologists, whether a lesion in the lungs is benign or malignant, is discussed logically, and the criteria of malignancy and benignity are listed. The role of CT is discussed. My only criticism of this section is the inclusion of primary lymphoma of the lung as a benign entity. Section 5 consists of 10 cases for self assessment.

The book is easily readable. The text is well written and concise, and I would recommend this book to radiologists in training, and also as a handy reference book for use in the radiological department.

R. J. JOHNSON